# One-Step Multiplex RT-qPCR Assay for the Detection of *Peste des petits ruminants virus*, *Capripoxvirus*, *Pasteurella multocida* and *Mycoplasma capricolum subspecies (ssp*.*) capripneumoniae*

**DOI:** 10.1371/journal.pone.0153688

**Published:** 2016-04-28

**Authors:** Tirumala Bharani Kumar Settypalli, Charles Euloge Lamien, Joachim Spergser, Mamadou Lelenta, Abel Wade, Esayas Gelaye, Angelika Loitsch, Germaine Minoungou, Francois Thiaucourt, Adama Diallo

**Affiliations:** 1 Animal Production and Health Laboratory (APHL), Joint FAO/IAEA Division of Nuclear Techniques in Food and Agriculture, Department of Nuclear Sciences and Applications, International Atomic Energy Agency (IAEA), Vienna, Austria; 2 Institute of Microbiology, University of Veterinary Medicine, Vienna, Austria; 3 Laboratoire National Vétérinaire (LANAVET), Annex Yaoundé, Cameroon; 4 Research and Diagnostic Laboratories, National Veterinary Institute, Debre Zeit, Ethiopia; 5 Institute for Veterinary Disease Control, Austrian Agency for Health and Food Safety, Mödling, Austria; 6 Laboratoire National d'Elevage, Ouagadougou, Burkina Faso; 7 Centre de Coopération Internationale en Recherche Agronomique pour le Développement (CIRAD), UMR CMAEE, Montpellier, France; University of Illinois at Urbana-Champaign, UNITED STATES

## Abstract

Respiratory infections, although showing common clinical symptoms like pneumonia, are caused by bacterial, viral or parasitic agents. These are often reported in sheep and goats populations and cause huge economic losses to the animal owners in developing countries. Detection of these diseases is routinely done using ELISA or microbiological methods which are being reinforced or replaced by molecular based detection methods including multiplex assays, where detection of different pathogens is carried out in a single reaction. In the present study, a one-step multiplex RT-qPCR assay was developed for simultaneous detection of Capripoxvirus (CaPV), Peste de petits ruminants virus (PPRV), *Pasteurella multocida* (PM) and *Mycoplasma capricolum* ssp. *capripneumonia* (Mccp) in pathological samples collected from small ruminants with respiratory disease symptoms. The test performed efficiently without any cross-amplification. The multiplex PCR efficiency was 98.31%, 95.48%, 102.77% and 91.46% whereas the singleplex efficiency was 93.43%, 98.82%, 102.55% and 92.0% for CaPV, PPRV, PM and Mccp, respectively. The correlation coefficient was greater than 0.99 for all the targets in both multiplex and singleplex. Based on cycle threshold values, intra and inter assay variability, ranged between the limits of 2%–4%, except for lower concentrations of Mccp. The detection limits at 95% confidence interval (CI) were 12, 163, 13 and 23 copies/reaction for CaPV, PPRV, PM and Mccp, respectively. The multiplex assay was able to detect CaPVs from all genotypes, PPRV from the four lineages, PM and Mccp without amplifying the other subspecies of mycoplasmas. The discriminating power of the assay was proven by accurate detection of the targeted pathogen (s) by screening 58 viral and bacterial isolates representing all four targeted pathogens. Furthermore, by screening 81 pathological samples collected from small ruminants showing respiratory disease symptoms, CaPV was detected in 17 samples, PPRV in 45, and PM in six samples. In addition, three samples showed a co-infection of PPRV and PM. Overall, the one-step multiplex RT-qPCR assay developed will be a valuable tool for rapid detection of individual and co-infections of the targeted pathogens with high specificity and sensitivity.

## Introduction

Small ruminants play a major role in the economic status of farmers from developing countries and are considered to be the ‘poor man’s’ financial resources. One of the major factors affecting the productivity of those animals is respiratory diseases caused by different types of pathogens: virus, bacteria and parasites in single or multiple infections [1–3]. The appropriate identification of the actual pathogen(s) responsible for the disease is critical for timely and proper management of those diseases.

In Asia, Africa and the Middle-East, major pathogens responsible for respiratory syndromes of sheep and goats, include *Capripoxvirus* (CaPV), *Peste des petits ruminants virus* (PPRV), *Pasteurella multocida* (PM) and *Mycoplasma capricolum* subspecies (ssp.) *capripneumoniae* (Mccp) [4–13]. Sheeppoxvirus and goatpoxvirus which belong to the CaPV genus are responsible for pox diseases in sheep and goats, diseases characterized by symptoms of fever, pox lesions over the skin and mucous membranes, conjunctivitis and sometimes respiratory distress [4–6]. PPRV, a negative-sense, single stranded RNA virus belonging to the morbillivirus group of the *Paramoxyviridae* family, causes an acute and highly infectious disease of small ruminants leading to high morbidity and mortality. It shares with goat and sheep pox nearly the same endemic regions [5], [7–8]. PM, a gram-negative bacterium, causes respiratory diseases in sheep, goats, pigs and cattle. It often causes pneumonia either alone or as an opportunist with other respiratory diseases causing pathogens of the upper respiratory tract [9–11]. Mccp is the causative agent of contagious caprine pleuropneumonia (CCPP) which causes significant economic losses to goat production in Africa and Asia. Although it is mainly known in goats, it has also been reported in sheep and a few wildlife species [12–14]. Because of the similarity in the symptoms caused by these specific pathogens, and their co-localization in nearly the same endemic areas, the proper management of these small ruminant respiratory diseases necessitates the implementation of rapid differential diagnostic tests for accurate identification of the responsible pathogen(s).

Multiplex molecular detection methods provide a good option for differential diagnosis and have the advantage of saving time and limiting the volume of sample requirement. In addition, surveillance using the multiplex testing of prevalent pathogens in high risk environments helps in proper management of animal diseases. Several investigators have previously developed PCR and real time PCR based multiplex assays for the differential detection of closely related pathogens or pathogens causing diseases with similar symptoms [15–18]. However, for the pathogens covered in this study, only singleplex PCR and real-time PCR detection methods are available. Hence the current study describes a single tube, one-step reaction for simultaneous detection of CaPV, PPRV, PM and Mccp causing respiratory infections in small ruminants.

## Materials and Methods

### Primers and probes design

The primers and probes were designed to amplify and detect specific regions for each of the four targeted pathogens. The targeted gene fragments were located on the 30 kDa RNA polymerase subunit (RPO30) gene for CaPV, the nucleoprotein (NP) gene for PPRV, the Kmt1 gene (alpha/beta hydrolase family protein) for PM and one helicase gene for Mccp. The full-length sequences of the targeted genes of PPRV, CaPV and PM were retrieved from GenBank and were aligned individually for each pathogen using MEGA Version 5.1. The target gene for Mccp was selected from the sequences generated by CIRAD, (Accession number: LM995445). Primers and probes were designed using the conserved regions of those genes using Allele ID® software version 6 (Premier Biosoft International, Palo Alto, USA). The specificity of the oligonucleotides was checked by using the NCBI-Primer BLAST tool (http://www.ncbi.nlm.nih.gov/tools/primer-blast/). The sequences of few of the primers and probes, were modified with degenerate bases, to cope with sequence variations. The hydrolysis probes were dually labelled with fluorescent dye (FAM/HEX/TEXAS RED/CY5) on the 5’ end and compatible black hole quencher (BHQ 1/2/3) on the 3’ end. Additional modifications included the incorporation of locked nucleic acids (LNA) in the probe sequences. The sequences of primers and probes, including the modifications, selected gene fragments of each pathogen, details of NCBI accession, nucleotide position and amplicon length are presented in Table 1. All primers and probes were manufactured by Eurogenetec (Seraing, Belgium).

**Table 1:** Primers and probes used for the amplification and detection of CaPV, PPRV, PM and Mccp. The different fluorescent labels on the 5’ end (FAM/HEX/TEXAS RED/CY5) and compatible black hole quencher on the 3’ end of the probes (BHQ 1/2/3) are displayed (LNAs are indicated by ‘+’ prior to the base). The targeted nucleotide sequence details are also provided.

**Oligo**	Sequences 5’-3’	Amplicon length	NCBI Accession	Nucleotide	
		(bp)	number	From	To
Capr_RPO_F	CTTCCAAGTTTTATATAAGAAA	*135*	GU119939.1	*134*	*268*
Capr_RPO_R	AAGGCTTGTTTCTATACGA				
N_Capr_RPO_Pb	*CY5*-CTTTT+GAGTAYT+CAATA+CCT-*BHQ3*				
PPR_N_F	CCATCAYTACCCGTTCAAG	*142*	X74443.2	*238*	*379*
PPR_N_R	ATYCGCTGKATCARTTGC				
N_PPR_N_Pb	*HEX*—GIG+ACT+CYACG+AACA- *BHQ1*				
PmtKmt_F	CAGAGTTTGGTGTGTTGA	*113*	DQ233648	*146*	*258*
PmtKmt_R	CAGACTGACAAGGAAATATAAAC				
PmtKmt_pb	*FAM* -AATC+TGC+TTCCTT+GAC- *BHQ1*				
Mccp_For	TTTTTCAAGTGCAAACGACTATG	*227*	LM995445	*741934*	*742160*
Mccp_Rev	TGACTTGGGTGTTAGGACCA				
Mccp_Pb_Tred	*TEXAS RED*-CGGATAG+AACAATA+GCTTTTACAGA- *BHQ2*				

+ indicates the LNA modification of the following base

### Nucleic acid extraction

Genomic DNA of PM, Mccp and other mycoplasma strains were extracted from cultures using the DNeasy Blood and Tissue kit (Qiagen, Valencia, CA, USA) at the source laboratories mentioned in supplementary information. The CaPV and PPRV isolates and pathological samples received from different sources (S1–S7 Tables) were processed at biosafety level 3 containment facility of the Institute for Veterinary Disease Control, Austria (HSL-AGES, Austria) and FAO/IAEA Animal Production and Health Laboratory (Seibersdorf, Austria). The total nucleic acid (TNA) extraction from tissue and cell culture lysates was performed using the High Pure PCR Template Preparation Kit (Roche Diagnostics GmbH, Mannheim, Germany) and RNeasy Mini Kit (Qiagen, Hilden, Germany) as per the manufacturers’ protocol with some modifications. The homogenized tissue supernatants or cell culture supernatants were prepared by adding Qiagen RLT plus lysis buffer (200 μL sample + 800 μL RLT plus buffer). The lysis buffer in the protocol was replaced by RLT plus buffer and on column DNAse digestion was avoided. The extracted TNA samples were stored at -70°C until further use.

### Optimization of the assay

Each pathogen specific assay was first optimized in singleplex to select the most appropriate annealing temperature, reagents, and primer and probe concentrations. The singleplex for CaPV, PM and Mccp detection included DNA as template whereas for PPRV detection included RNA. As the multiplex assay targeted the combination of RNA and DNA pathogens, the multiplex assay conditions were modified accordingly and were evaluated for RT-PCR conditions. The optimized conditions for the one-step multiplex RT-qPCR assay were as follows: 2 μL of nucleic acid added to a reaction mixture of 1X iScript universal probes reaction mix and 1X iScript advanced RT enzyme from iScript™ Universal Probes One-Step Kit (Bio-Rad Laboratories, Hercules, USA), 500 nM of each primer and 250 nM of each probe in the final reaction volume of 20 μL. The one-step multiplex RT-qPCR was developed and evaluated on the CFX 96™ real time PCR machine (Bio-Rad) with cycling parameters as follows: 50°C for 20 min followed by 95°C for 5 min and 40 cycles of denaturation at 94°C for 10 sec, annealing at 56°C for 20 sec, and extension at 62°C for 20 sec. The data acquisition was performed during the annealing step on four different channels: green (FAM), yellow (HEX), orange (TEXAS RED) and red (CY5).

### Positive controls

#### Construction of plasmids

A plasmid containing the full-length RPO30 gene sequence of the sheeppox virus (SPPV) Denizli [19] was available in the laboratory and was used for the CaPV positive control. The genomic DNA of PM and Mccp were subjected to PCR amplification using the respective primers specified in Table 1. The obtained amplicons were then cloned into pGEM-T plasmid (Promega, Madison, WI, USA), and the identity of each insert was checked by sequencing. The concentration of the plasmid was estimated spectrofluorometrically using Quant-iT™ PicoGreen® dsDNA Assay Kit (Invitrogen, Eugene, OR, USA) and the copy number was determined following the steps described previously [19]. For each pathogen, except for PPRV, the corresponding plasmids were serially diluted 10-fold from 10^8^ to 10^1^ copies/μL and were used to determine the analytical sensitivity.

#### Synthesis of PPRV RNA transcripts

For PPRV, the full NP gene of 1,692 bp size was amplified and cloned into plasmid pCI-neo (Promega,), downstream of the T7 promoter region. The recombinant plasmid was linearized by digesting with *Not I* (New England Bio Labs, Ipswich, MA, USA) and was purified using Wizard DNA Clean-Up System (Promega). An *in vitro* transcription with T7 RNA polymerase and the DNA template removal was carried out using the RiboMax^TM^ Large Scale RNA Production System (Promega). The absence of DNA traces was confirmed by amplifying the transcripts without reverse transcriptase (RT) enzyme. The transcript was quantified using synthetic RNA oligo with known concentration as the standard (to be published elsewhere). The RNA transcripts were further diluted for the evaluation of analytical sensitivity. The three plasmids, each with a respective gene fragment for CaPV, PM or Mccp and RNA transcripts of PPRV were further used to study the analytical validation of the assay and were included as positive controls for further runs.

### Linearity

The efficiency and dynamic range of the multiplex assay and the respective singleplex assays were established by amplifying eight 10-fold serial dilutions from 10^8^ to 10^1^ of plasmid containing the RPO30 gene of CaPVs, Kmt1 gene of PM and helicase gene of Mccp and 10-fold serial dilutions from 5 x 10^6^ to 5 x 10^1^ of RNA transcripts of the NP gene of PPRV. Each dilution was amplified in triplicate during three separate runs.

### Analytical sensitivity of the assay

The limit of detection of the multiplex assay was determined for each pathogen as follows: for CaPV and PM, plasmid dilutions of 20, 16, 12, 8 and 4 copies/reaction were assayed. For Mccp, the dilutions were: 80, 40, 16, 12, 8 and 4 copies/reaction and PPRV transcript dilutions were 200, 160, 120, 80 and 40 copies/reaction. The analytical sensitivity of the assay was determined by testing these diluted plasmids and PPRV transcripts for amplification in pentaplicate separately at three different intervals. The proportion of predictive positive results was determined by regression analysis using the STATGRAPHICS Centurion XV Version 15.2.12 software package (StatPoint Technologies, Warrenton, VA, USA).

### Repeatability

Intra- and inter-assay variability for the multiplex assay were determined at high, medium and low copy number (10^7^, 10^5^ and 10^3^) using the threshold values obtained from the quantification cycle (Cq) generated by amplification of diluted plasmids for CaPV, PM and Mccp and diluted transcripts for PPRV. The test was performed in triplicate for the selected dilutions and at three different intervals.

### Specificity and detection power of the assay

The specificity and detection power of the assay were evaluated using nucleic acid extracted from cultured isolates and clinical samples. DNA samples were extracted from 20 CaPV positives isolates (SPPV and GTPV), 21 PM positive isolates, seven Mccp isolates and RNA samples were extracted from 10 PPRV isolates belonging to four different lineages. The samples tested for each targeted pathogen were collected from different geographical locations. The details of the samples *i*.*e*., sample source, sample type and result using the multiplex RT-qPCR assay are summarised in supplementary information (S1–S4 Tables). The assay specificity was further determined by attempting to amplify nucleic acid from non-specific pathogens like Bovine respiratory syncytial virus (BRSV), Poxvirus (BPSV and Orf virus), Parainfluenza virus 3 (PIV3) and other mycoplasma species including *M*. *putrefaciens*, *M*. *ovipneumoniae*, *M*. *mycoides* ssp. *mycoides* (SC- small colony type), *M*. *mycoides* ssp. *capri* (including former *M*. *mycoides* ssp. *mycoides* (LC—large colony type), *M*. *leachii*, *M*. *conjunctivae*, *M*. *capricolum* ssp. *capricolum*, *M*. *bovis*, and *M*. *agalactiae* (S5 Table).

The detection power and usability of the multiplex one-step RT-qPCR assay were also evaluated by screening nucleic acid extracts from swab and tissue samples collected from sheep/goats suspected for Peste des petits ruminants (PPR) or Capripox and having respiratory disease symptoms. This included 58 TNA samples and 23 DNA samples (S6 and S7 Tables). The method was also evaluated at the Laboratorie National d’Elevage, Burkina Faso using 13 denatured swab samples collected from small ruminants suspected with PPRV infection. These 13 samples were tested again using the TNA extractions.

The ability of the assay to detect Lumpy skin disease virus (LSDV), a genotype of CaPV infecting cattle, was tested by screening 60 DNA samples extracted from clinical samples collected on diseased cattle in Ethiopia, Sudan and Democratic Republic of Congo (S8 Table). For all clinical samples, the obtained results were further validated by using previously established classical PCR and real time PCR methods (S9 Table) [20–23].

## Results

### Assay design and optimization

**Fig 1:**
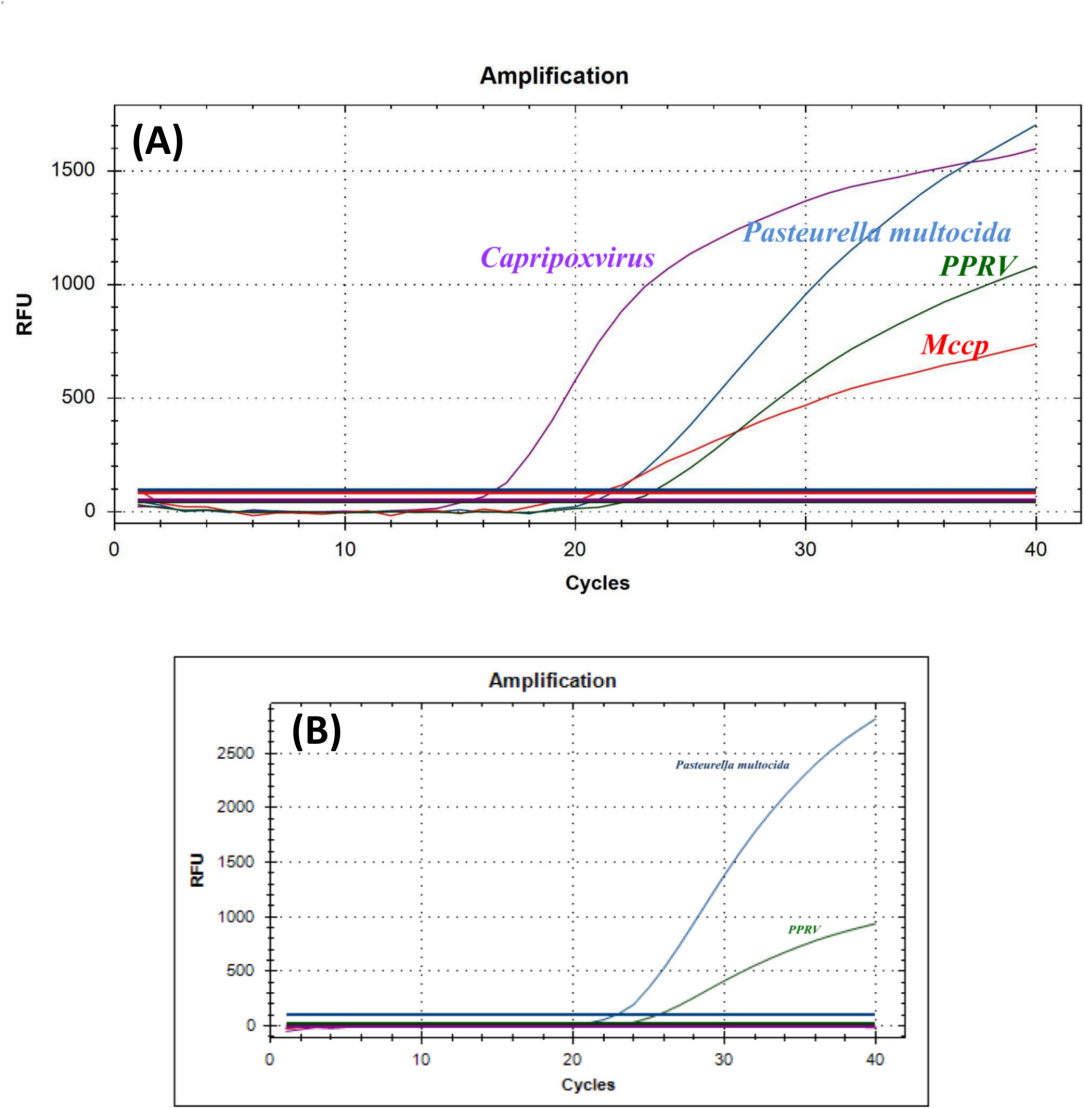
Amplification patterns obtained using the One-step multiplex RT-PCR. **(A) Simultaneous detection of CaPV, PPRV, PM and Mccp in a single tube:** Amplification curves of three separate plasmids harbouring three selected gene fragments and of RNA transcripts of PPRV are shown. The amplification curves are shown for CaPV (Cyan), PM (Blue), Mccp (Red) and PPRV (Green). The curves were generated by the amplification of a pooled control sample at a final concentration of 10^5^ copies /μL of PPRV, PM and Mccp controls and 10^6^ copies /μL of CaPV control. **(B) Simultaneous detection of mixed infection while screening the swab samples of infected sheep at Burkina Faso**. Two amplification curves of FAM-Blue and HEX-Green colours indicate the clear presence of dual infection of PM and PPRV respectively. The absence of TEXAS RED-Red and CY5-Cyan coloured peaks or background indicate the absence of Mccp and CaPV infections, respectively.

The primers and probes were modified during assay development by incorporating degenerate bases in order to detect all variants of CaPV genotypes, PPRV, PM and Mccp. Modification of probes was done by adding LNAs internally which allowed the short length of probes, their alignment with the multiple sequences used in the design, and to maintain a high annealing temperature. The probes were labelled with a different fluorescent reporter on each of the 5’ ends and a compatible quencher on the 3’ ends (Table 1). The assay detected all four targeted pathogens without any cross-amplification and non-specific background using the optimized PCR conditions. The amplification patterns of the target sequences of each pathogen were clear and are indicated by an amplification curve with colour representing the probe labelled with respective dye (Fig 1A and 1B). Non-specific background fluorescence or signal interference was not observed throughout the present study.

### Linearity and analytical sensitivity of the assay

**Fig 2:**
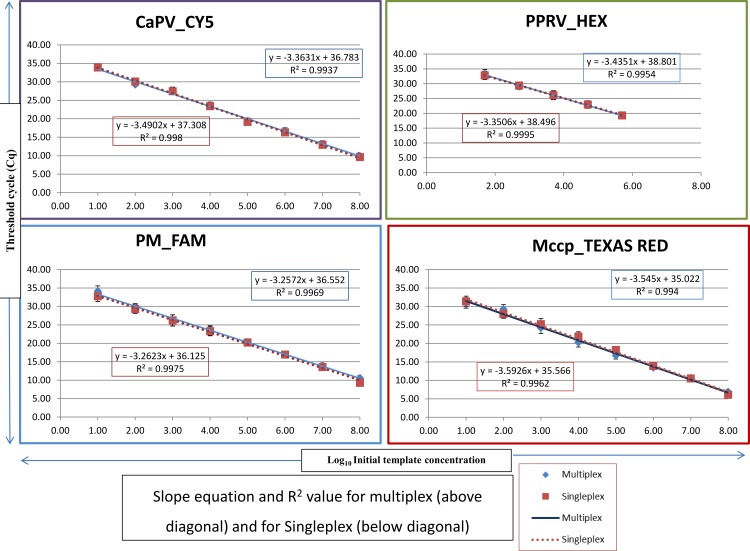
Linearity of multiplex and singleplex assays. The calibration curves were generated by amplification of 10-fold dilutions of plasmid DNA standards for CaPV, PM, Mccp and RNA transcripts of PPRV using one-step multiplex RT-qPCR and singleplex RT-qPCR for respective target. Each dilution was run in triplicate at three different intervals. The mean Cq values are plotted against the log of concentration of the target gene copies/reaction. The PCR efficiency (***E***) for each target was calculated using the slope of each calibration curve with the formula ***E*** = 10^−1/slope^−1 was greater than 90% and the correlation coefficient (R^2^) was greater than 0.99.

The linearity of the assay was established by amplifying 10-fold serial dilutions (10^8^ to 10^1^ copies/reaction) of plasmid containing the RPO30 gene of CaPV, Kmt1 gene of PM, helicase gene of Mccp and RNA transcripts of NP gene of PPRV. The resulting Cq values were plotted against the log input copy number. The linearity of the multiplex assay ranged from 10^8^ to 10^2^, 10^6^ to 10^2^, 10^8^ to 10^1^ and 10^7^ to 10^1^ with efficiency of 98.31%, 95.48%, 102.77% and 91.46% for CaPV, PPRV, PM and Mccp, respectively with a correlation coefficient (R^2^) greater than 0.990 (Fig 2). The linearity of singleplex PCR for each targeted pathogen was similar to that of multiplex with an efficiency of 93.43%, 98.82%, 102.55% and 92.0% for CaPV, PPRV, PM and Mccp respectively and a R^2^ value greater than 0.990 (Fig 2).

The limit of detection of the multiplex assay was determined by probit regression analysis using the observed proportion of positive results from the amplification of 5 different concentrations of plasmid and transcript of target gene assayed in replicates of five in five different runs. The limits of detection at a ≥95% chance for copies of each pathogen were 11.95 (9.9 to 15.5), 162.44 (138.2 to 204.7), 12.05 (10.1 to 15.4) and 22.21 (8.13 to 30.2) copies per reaction for CaPV, PPRV, PM and Mccp, respectively.

### Inter- and intra-assay variability

The reproducibility of the multiplex assay was assessed between and within runs. Three different concentrations of each plasmid of CaPV, PM and Mccp, two different concentrations of RNA transcripts of PPRV were assayed in triplicate in three separate runs that were performed on different days. The coefficients of variation between and within runs are summarized in Table 2. The coefficients of variation between the runs were mostly in the range of 1.422 to 3.68 except for higher and lower concentration of Mccp (6.05 and 4.15, respectively).

**Table 2:** Inter- and Intra-assay variability of the one-step multiplex RT-qPCR assay. The variability was calculated based on the threshold values for the amplification of three different concentrations of controls—higher (10^7^), medium (10^5^) and lower concentrations (10^3^) of each targeted pathogen run at three different intervals.

		Inter-	Intra-assay variability (by Cq Values)		
	Template Concentration	assay variability (by Cq values)	Run1	Run2	Run3
**CaPV**	High (10^7^)	2.620	0.525	3.692	0.571
	Medium (10^5^)	2.418	2.348	0.246	0.473
	Low (10^3^)	2.510	0.702	2.118	0.292
**PPRV**	High (10^7^)	ND	ND	ND	ND
	Medium (10^5^)	2.688	2.113	0.267	0.955
	Low (10^3^)	2.380	1.743	0.630	0.781
**PM**	High (10^7^)	2.884	1.249	0.339	1.249
	Medium (10^5^)	1.422	0.714	0.977	0.716
	Low (10^3^)	2.224	0.416	0.484	0.299
**Mccp**	High (10^7^)	6.05	1.94	1.64	2.54
	Medium (10^5^)	3.68	3.21	1.60	3.67
	Low (10^3^)	4.15	1.95	1.74	0.48

### Specificity and assay performance

To demonstrate the specificity and performance of the one-step multiplex RT-qPCR, nucleic acids from known positive samples of CaPV, PPRV, PM and Mccp were tested. Twenty isolates of known CaPVs (SPPV and GTPV) tested positive for CaPV using the multiplex assay, indicating a 100% agreement with previously established assays. Similarly, all ten PPRV isolates belonging to four different lineages were detected as PPRV in the multiplex assay and 21 positive PM isolates were all detected as PM in the multiplex assay giving a specificity of 100%. The detection of Mccp was very specific by amplifying all seven Mccp isolates tested. While amplifying these different isolates of four targeted pathogens, there was neither false positivity nor significant interference between the four fluorophore signals, indicating a high specificity. The assay was also highly specific to the pathogens intended to be detected as no amplification was observed with untargeted pathogens such as BRSV, Orf virus, Parainfluenza virus (PIV3) and other mycoplasma species (S5 Table).

Further evaluation was done by screening 81 nucleic acid samples (58 TNA and 23 DNA samples) extracted from various types of specimens collected from small ruminants with clinical signs of respiratory diseases (S6 and S7 Tables). Results from those 81 samples tested, 17 were positive for CaPV, 45 for PPRV, 7 for PM and none for Mccp. Mixed infection of PPRV and PM was observed in 3 TNA samples. The summary of isolates and samples screened during the development and evaluation of the test is mentioned in Table 3. For all positive samples, the results were further confirmed using a respective confirmatory test (primers in S9 Table).

**Table 3:** Summary of viral/bacterial isolates and field samples screened by one-step multiplex RT-qPCR. The positivity was further confirmed by the respective confirmatory tests.

	Total number of samples	CaPV	PPRV	PM	Mccp	None
Confirmed isolates/samples	n = 58	20	10	21	7	NA
DNA of suspected samples	n = 23	17	NA	1	0	5
TNA of suspected samples	n = 58	0	45(3)^δ^	6(3)^δ^	0	10
Tota1	139	37	55	28	7	15

^δ^ Three samples mentioned in parenthesis indicate the co-infection of PM and PPRV

The majority of the screened samples tested was positive for PPRV (55.5%) followed by CaPV (20.9%). The sample KN19\2011, from Kenya with suspicion of PPR infection, was positive for PM and negative for PPRV when tested using our one-step multiplex RT-qPCR. The sample was confirmed to be negative for PPRV when tested using the classical PCR [21]. Of the 13 swab samples tested at the Laboratoire National d’Elevage, Burkina Faso, seven samples were positive for PPRV, five for PM and three were positive for both PPRV and PM also indicating the co-infection. Out of 60 DNA extract from pathological tissues of cattle suspected with respiratory disease, 31 samples were positive for CaPV. These were further confirmed to be of LSDV genotype using a CaPV species specific detection method.

## Discussion

The one-step multiplex RT-qPCR assay was developed for detection of two bacterial and two viral pathogens known to cause respiratory infections in small ruminants. The assay was as sensitive as singleplex PCR assays and very specific, detecting all three genotypes of CaPV, isolates in each of the four lineages of PPRV, PM and Mccp. As the present method targets RNA virus, DNA virus and bacteria simultaneously, the extraction of total nucleic acids is considered to be one of the crucial steps. The High Pure PCR Template Preparation Kit (Roche) was validated earlier for DNA and RNA extraction from African swine fever virus and classical swine fever virus respectively [24]. We evaluated the Qiagen RNeasy Mini Kit along with the former mentioned kit for the extraction of TNA from samples known to be positive for one and/or two targeted pathogens. During our study, the TNA extraction was more efficient when using a modified protocol of the RNeasy mini kit (S10 Table). This may be due to our usage of RLT plus buffer for lysis in place of the recommended RLT buffer and avoiding the step of DNase digestion. Further validation is needed by testing extraction from different sample matrices and a larger subset of samples. For the amplification of RNA, two step reactions- cDNA synthesis and amplification are combined by using single tube one-step RT-PCR which further eliminates the usage of two different kits and lowers the chance of contamination.

Multiplex tests for detection of different pathogens in a single reaction can aid in a more rapid diagnosis but does present a few disadvantages such as complexity in handling the protocol and the requirement of advanced technologies: microarrays, liquid arrays, and mass spectrophotometers. Real time PCR when used with fluorescent probes allows for multiplexing with ease of handling and similar efficiency as the singleplex assay. The cost of the probes and the complexity involved in the proper optimization of multiplex PCR can be overcome by the overall advantages of the multiplex assay. Advantages include, screening more target pathogens with less sample volume, reducing laboratory testing time and resources. Moreover it is highly useful tool for disease surveillance by screening large numbers of samples.

The sensitivity of this multiplex assay was similar to previously published singleplex assays for all the pathogens. Lamien *et al*., [19] and Gelaye *et al*., [25] were able to detect 250 copies and 20 copies of CaPV by using the classical PCR and real time PCR methods respectively. Bao *et al*., [26] and Kwiatek *et al*., [27] achieved lower detection limit of 8.1 and 32 RNA copies of PPRV using singleplex real time PCR methods. As the LOD of PPRV detection (163 copies / reaction) appeared to be higher than that of previously reported assays [26–27], we have undertaken a study to compare these two methods to our current multiplex assay. Using serial dilutions of our RNA transcript the sensitivity of one-step multiplex RT-PCR for PPRV detection was similar to Bao *et al*., [26] method which detected 200 copies / reaction, and higher than Kwiatek *et al*., [27] method which detected 300 copies / reaction. Since these values are much higher than that of the LODs observed by these authors, we suspected that the stringency in the purification of our PPRV transcripts and the differences in the quantitation methods followed by various laboratories might have resulted the observed differences. To further investigate on the similarity in the sensitivities of the two singleplex assays and our multiplex assay in PPRV detection, we have additionally tested serial dilutions of two TNA extracts using the three methods. The results showed almost similar amplification pattern by all the three PPRV detection methods as indicated by Cq values (S11 Table).

The inter- and intra-assay variability indicates the performance consistency of all primers and probes during different runs of the multiplex assay with an optimized annealing temperature. Overall the inter- and intra-assay variability was low except for Mccp. Although the assay was proven to be very specific for CaPV, PPRV and Mccp, more variants of PM need to be evaluated. This multiplex RT-qPCR specificity was established by comparing its accuracy to previously established methods. There was complete agreement between the current assay and previously established methods for CaPV [20] PPRV [21], PM [22] and Mccp [23]. Additionally, only Mccp isolates were amplified and detected, when tested against different mycoplasma species and subspecies. Specific detection of Mccp is challenging due to the existence in goats of several mycoplasma species which are phylogenetically similar. Mccp specificity was achieved by selection of a target gene which is strictly specific to Mccp strains. The high specificity of Mccp detection indicates the flexibility of usage of these primers and probes in singleplex for Mccp detection if needed to screen CCPP during surveillance or outbreaks. During this study, while testing the clinical samples from different geographical locations, the multi-plex test efficiently detected all the pathogens present either individually or as a co-infection. The number of CaPV and PPRV positives is high because the field samples tested were suspected of containing infection of Capripox or PPR based on clinical symptoms. The PPRV suspected sample from Kenya (KN19\2011), was confirmed negative for PPRV by both the one-step multiplex RT-qPCR test and the confirmatory classical PCR test [21]. With the developed multiplex assay, the same sample was detected to be positive for PM. These results indicate the high accuracy, specificity and performance of the assay for the detection of four targeted pathogens and can rule out the false suspicions. During testing for simultaneous detection of all four templates in a single tube using pooled controls, the detection level of CaPV was very low due to a reduction in the RFU values compared to the other template-probes. When testing the pooled template with both the multiplex and singleplex assay using a range of dilutions of 10^5^ to 10^1^, it was observed that each individual template produced a similar Cq value for both assays with reduced RFU values in the multiplex. This may be due to increased competition occurring when trying to amplify all four templates at one time and may not hinder the efficiency when only two or three targets are present in the real time samples (Fig 1B).

Of thirteen swab samples (BKF02/201404 to BKF14/201404) suspected for respiratory infections, screened at Laboratoire National d’Elevage, Burkina Faso, 7 of them were positive for PPRV and 5 with PM. Of the 7 positive PPRV samples, 3 of them were also positive for PM. The detection of dual infection of PPRV and PM is shown in Fig 1B. The assay in that laboratory was performed using directly heat inactivated swab samples without nucleic acid extraction. The results were later confirmed by performing the test with extracted TNA. This demonstrates the capability of the test to accurately detect the pathogens, and shows the flexibility of the protocol, when used in developing countries, by allowing the direct use of swab samples without TNA extraction. The usage of swab samples for amplification without nucleic acid extraction needs to be further evaluated with additional samples and in different laboratories.

The one-step multiplex RT-qPCR was able to confirm the prevalence of co-infections of PPRV and PM among the field samples tested at Burkina Faso. Among the few other samples tested during development (data not shown), cases of mixed infections of CaPV and PPRV, PPRV and PM were encountered. Overall, this study demonstrated the capability of a multiplex assay to detect individual and co-infections of the targeted pathogens. This information will allow for rapid implementation of appropriate actions for the effective control of the disease and its spread. The multiplex assay also provides the opportunity to detect pathogens in previously unknown hosts. This allows for applicable use of the assay in surveillance of either domestic and/or wildlife samples where there is limited sample volume. In April 2015, at an international conference that was organized by the Food and Agriculture Organisation of the United Nation (FAO) and the World Organisation for Animal Health (OIE) on PPR control, these two international organizations presented a strategy for the global control and eradication of this disease. The strategy that was endorsed by the participants includes the control of other priority small ruminant diseases such as CCPP and Capripox. At the later stage of the eradication programme, respiratory syndromic cases in sheep and goats will be investigated for residual PPR infection. For that purpose, this one-step multiplex RT-qPCR for the identification of four pathogens responsible for respiratory diseases of small ruminants will be a useful tool for disease surveillance. Additionally, other pathogens responsible for respiratory diseases of small ruminants need to be included in the multiplex based tests. In the meantime, preliminary results obtained in this study will undergo further evaluation by testing additional samples, healthy and infected, to establish it as a routine and efficient surveillance diagnostic tool.

## Supporting Information

S1 Table Details of the DNA samples extracted from CaPV (SPPV/GTPV) isolates and results on testing by one-step multiplex RT-qPCR which were further confirmed by snapback real time PCR [20]. (DOC)

S2 Table Details of the RNA samples extracted from four different lineages of confirmed PPRV infected cell culture or tissue samples and results on testing by one-step multiplex RT-qPCR which were further confirmed by classical PCR [21]. (DOC)

S3 Table Details of the DNA samples extracted from *Pasteurella multocida* (PM) isolates and results on testing by one-step multiplex RT-qPCR which were further confirmed by classical PCR [22]. (DOC)

S4 Table Details of the DNA samples extracted from different mycoplasma species and isolates of Mccp and results on testing by one-step multiplex RT-qPCR which were further confirmed by classical PCR [23]. (DOC)

S5 Table Details of the non-specific pathogens tested using the one-step multiplex RT-qPCR. (DOC)

S6 Table Details of the TNA samples extracted from different field samples collected from Goat and Sheep showing the respiratory infections (mainly suspected for PPRV) and results on testing by one-step multiplex RT-qPCR. (DOC)

S7 Table Details of the DNA samples extracted from different pathological samples collected from Goat and Sheep showing the symptoms of respiratory infections and results on testing by one-step multiplex RT-qPCR. (DOC)

S8 Table Details of the DNA samples extracted from different pathological samples collected from cattle showing the symptoms of respiratory infections and results on testing by one-step multiplex RT-qPCR. (DOC)

S9 Table Primer sequences and reference used for the confirmation of four targeted pathogens-CaPV, PPRV, PM and Mccp. (DOC)

S10 Table Performance of the multiplex assay in detection of TNA of different targets (represented by Cq values) extracted using two different extraction kits. (DOC)

S11 Table Comparison of performance of multiplex assay for detection of PPRV with two previously described methods. (DOC)
