# Supplementary material for: One-Step Multiplex RT-qPCR Assay for the Detection of Peste des petits ruminants virus, Capripoxvirus, Pasteurella multocida and Mycoplasma capricolum subspecies (ssp.) capripneumoniae
Source: PLoS One. 2016 Apr 28;11(4):e0153688. doi: 10.1371/journal.pone.0153688 (PMC4849753; doi:10.1371/journal.pone.0153688)
Supplement: S1 Table — (DOC) [file pone.0153688.s001.doc]

**Table S1: Details of the DNA samples extracted from CaPV (SPPV/GTPV) isolates and results on testing by one-step multiplex RT-qPCR which were further confirmed by snapback real time PCR [16]**

| **S No** | **Sample ID** | **Origin** | **Species/genotype/** | **Test results/Detected pathogen(s)** | **Received from** | **Sample type** |
| --- | --- | --- | --- | --- | --- | --- |
|  | SPPV Algerie/05 Illizi | Algeria | SPPV | Positive for *CaPV* | INMV-LCV, Algeria | Cell culture |
|  | SPPV Algerie/93 Djelfa | Algeria | SPPV | Positive for *CaPV* | INMV-LCV, Algeria | Cell culture |
|  | SPPV Algeria Vaccine | Romania | SPPV | Positive for *CaPV* | INMV-LCV, Algeria | Cell culture |
|  | GTPV Bangladesh/86 | Bangladesh | GTPV | Positive for *CaPV* | IAH-Pirbright, UK | Cell culture |
|  | GTPV Ghana | Ghana | GTPV | Positive for *CaPV* | IAH-Pirbright, UK | Cell culture |
|  | GTPV Iraq/61 Gorgan | Iraq | GTPV | Positive for *CaPV* | IAH-Pirbright, UK | Cell culture |
|  | SPPV vaccine Morocco | Morocco | SPPV | Positive for *CaPV* | Biopharma, Morocco | Cell culture |
|  | GTPV Oman/84 | Oman | GTPV | Positive for *CaPV* | IAH-Pirbright, UK | Cell culture |
|  | SPPV Oman | Oman | SPPV | Positive for *CaPV* | IAH-Pirbright, UK | Cell culture |
|  | SPPV Oman | Oman | SPPV | Positive for *CaPV* | IAH-Pirbright, UK | Cell culture |
|  | GTPV Saudi Arabia/93 | Saudi Arabia | GTPV | Positive for *CaPV* | IAH-Pirbright, UK | Cell culture |
|  | GTPV Turkey/98 Denizli | Turkey | GTPV | Positive for *CaPV* | VCRI-Pendik, Turkey | Cell culture |
|  | SPPV Turkey/98 Darica | Turkey | SPPV | Positive for *CaPV* | VCRI-Pendik, Turkey | Cell culture |
|  | SPPV Turkey/98 Denizli | Turkey | SPPV | Positive for *CaPV* | VCRI-Pendik, Turkey | Cell culture |
|  | SPPV Turkey/98 Sivas | Turkey | SPPV | Positive for *CaPV* | VCRI-Pendik, Turkey | Cell culture |
|  | SPPV Turkey/98 Van 2 | Turkey | SPPV | Positive for *CaPV* | VCRI-Pendik, Turkey | Cell culture |
|  | SPPV Turkey/98 Çorum | Turkey | SPPV | Positive for *CaPV* | VCRI-Pendik, Turkey | Cell culture |
|  | SPPV HSL | Unknown | SPPV | Positive for *CaPV* | HSL-AGES, Austria | Cell culture |
|  | SPPV Soba | Sudan | SPPV | Positive for *CaPV* | CVRL, Sudan | Cell culture |
|  | GTPV Yemen | Yemen | GTPV | Positive for *CaPV* | IAH-Pirbright, UK | Cell culture |

*INMV-LCV- Institut National de la Médecine Vétérinaire, Locaux administratifs et Laboratoire Central Vétérinaire; IAH-Institute for animal health, Pirbright; VCRI- Veterinary Control and Research Institutes, Pendik; HSL-AGES- High Security Laboratory, Austrian Agency for Health and Food Safety, Moedling; CVRL-Central veterinary research Laboratories.*
