# Supplementary material for: One-Step Multiplex RT-qPCR Assay for the Detection of Peste des petits ruminants virus, Capripoxvirus, Pasteurella multocida and Mycoplasma capricolum subspecies (ssp.) capripneumoniae
Source: PLoS One. 2016 Apr 28;11(4):e0153688. doi: 10.1371/journal.pone.0153688 (PMC4849753; doi:10.1371/journal.pone.0153688)
Supplement: S2 Table — (DOC) [file pone.0153688.s002.doc]

**Table S2:** Details of the RNA samples extracted from four different lineages of confirmed PPRV infected cell culture or tissue samples and results on testing by one-step multiplex RT-qPCR which were further confirmed by classical PCR [17]

| **S No** | **Sample ID** | **Origin** | **Lineage** | **Multiplex result & Detected pathogen(s)** | **Received from** | **Sample type** |
| --- | --- | --- | --- | --- | --- | --- |
|  | Guinea PPR Vero1 CHS1\1992 | Guinea | I | Positive for PPRV | CIRAD, France | Virus isolate |
|  | E32\1969 | Senegal | I | Positive for PPRV | ISRA, Senegal | Tissue |
|  | Ngith\1970 | Senegal | I | Positive for PPRV | ISRA, Senegal | Tissue |
|  | Ghana PPR CHS1\2011 | Ghana | II | Positive for PPRV | CVL, Ghana | Virus isolate |
|  | Benin\1969 | Benin | II | Positive for PPRV | ISRA, Senegal | Tissue |
|  | Kenya PPR CHS1\2011 | Kenya | III | Positive for PPRV | CVL, Kenya | Virus isolate |
|  | Dorcas | Oman | III | Positive for PPRV | IAH-Pirbright, UK | Virus isolate |
|  | Mielik\1972 | Sudan | III | Positive for PPRV | IAH-Pirbright, UK | Virus isolate |
|  | Sinnar\1972 | Sudan | III | Positive for PPRV | IAH-Pirbright, UK | Virus isolate |
|  | Pakistan PPR CHS1\2010 | Pakistan | IV | Positive for PPRV | NIBGE, Pakistan | Virus isolate |

*CIRAD-Centre International de Rechercheen Agronomie pour le Développement; ISRA*- *Institut Sénégalais de Recherche Agricole; CVL- Central veterinary laboratorie; CVL*- *Central Veterinary laboratories*; *IAH-Institute for animal health, Pirbright, UK; NIBGE -National Institute of Biotechnology & Genetic Engineering, Faisalabad.*
