# Supplementary material for: One-Step Multiplex RT-qPCR Assay for the Detection of Peste des petits ruminants virus, Capripoxvirus, Pasteurella multocida and Mycoplasma capricolum subspecies (ssp.) capripneumoniae
Source: PLoS One. 2016 Apr 28;11(4):e0153688. doi: 10.1371/journal.pone.0153688 (PMC4849753; doi:10.1371/journal.pone.0153688)
Supplement: S3 Table — (DOC) [file pone.0153688.s003.doc]

**Table S3:** Details of the DNA samples extracted from *Pasteurella multocida* (PM) isolates and results on testing by one-step multiplex RT-qPCR which were further confirmed by classical PCR [18]

| **S No** | **Sample ID** | **Origin** | **Multiplex result & Detected pathogen(s)** | **Received from** | **Sample type** |
| --- | --- | --- | --- | --- | --- |
|  | B9 | Cameroon | Positive for PM | LANAVET, Cameroon | Microbial Culture |
|  | E2 | Cameroon | Positive for PM | LANAVET, Cameroon | Microbial Culture |
|  | G2 | Cameroon | Positive for PM | LANAVET, Cameroon | Microbial Culture |
|  | IR 2 - GAROUA | Cameroon | Positive for PM | LANAVET, Cameroon | Microbial Culture |
|  | 6 GOULMONA | Cameroon | Positive for PM | LANAVET, Cameroon | Microbial Culture |
|  | B6 | Cameroon | Positive for PM | LANAVET, Cameroon | Microbial Culture |
|  | B2 | Cameroon | Positive for PM | LANAVET, Cameroon | Microbial Culture |
|  | E2(9/12/2009) | Cameroon | Positive for PM | LANAVET, Cameroon | Microbial Culture |
|  | 97-FED(19/06/93) | Cameroon | Positive for PM | LANAVET, Cameroon | Microbial Culture |
|  | 102-FED (01/01/93) | Cameroon | Positive for PM | LANAVET, Cameroon | Microbial Culture |
|  | B62 (13/11/1996) | Cameroon | Positive for PM | LANAVET, Cameroon | Microbial Culture |
|  | E63 (13/11/1996 | Cameroon | Positive for PM | LANAVET, Cameroon | Microbial Culture |
|  | E 62 Makari (30/05/96) | Cameroon | Positive for PM | LANAVET, Cameroon | Microbial Culture |
|  | G1 E2 16/07/2004) | Cameroon | Positive for PM | LANAVET, Cameroon | Microbial Culture |
|  | E6 1 GOULMOUN (30/05/96) | Cameroon | Positive for PM | LANAVET, Cameroon | Microbial Culture |
|  | PM5 | Mongolia | Positive for PM | IVM, Mongolia | Microbial Culture |
|  | PM6 | Mongolia | Positive for PM | IVM, Mongolia | Microbial Culture |
|  | Ovine Pas Mol A 01 | Ethiopia | Positive for PM | NVI, Ethiopia | Microbial Culture |
|  | Ovine Pas Mol A 02 | Ethiopia | Positive for PM | NVI, Ethiopia | Microbial Culture |
|  | TFY_PA1 | Ethiopia | Positive for PM | NAHDIC, Ethiopia | Microbial Culture |
|  | TFY_PA2 | Ethiopia | Positive for PM | NAHDIC, Ethiopia | Microbial Culture |

*LANAVET - Laboratoire National Vétérinaire; IVM***-***Institute of Veterinary Medicine; NVI- National Veterinary Institute; NAHDIC- National Animal Health Diagnostic and Investigation Center.*
