# Supplementary material for: One-Step Multiplex RT-qPCR Assay for the Detection of Peste des petits ruminants virus, Capripoxvirus, Pasteurella multocida and Mycoplasma capricolum subspecies (ssp.) capripneumoniae
Source: PLoS One. 2016 Apr 28;11(4):e0153688. doi: 10.1371/journal.pone.0153688 (PMC4849753; doi:10.1371/journal.pone.0153688)
Supplement: S4 Table — (DOC) [file pone.0153688.s004.doc]

**Table S4:** Details of the DNA samples extracted from different mycoplasma species and isolates of Mccp and results on testing by one-step multiplex RT-qPCR which were further confirmed by classical PCR [19]

| **S No** | **Sample ID** | **Origin** | **Species/genotype/** | **Multiplex result & Detected pathogen(s)** | **Received from** | **Sample type** |
| --- | --- | --- | --- | --- | --- | --- |
|  | A1\Mccp\Abomsa\ET | Ethiopia | *Mccp* | Positive for Mccp | *CIRAD, France* | Microbial Culture |
|  | *95043\1995* | Niger | *Mccp* | Positive for Mccp | *CIRAD, France* | Microbial Culture |
|  | *98113* | Uganda | *Mccp* | Positive for Mccp | *CIRAD, France* | Microbial Culture |
|  | *12002* | Tajikistan | *Mccp* | Positive for Mccp | *CIRAD, France* | Microbial Culture |
|  | *Mccp F38^T^* | Kenya | *Mccp* | Positive for Mccp | *Vetmed, Vienna* | Microbial Culture |
|  | Mccp\2012-11\ ETH01 | Ethiopia | *Mccp* | Positive for Mccp | *NVI, Ethiopia* | Microbial Culture |
|  | Mccp \2012-11\ ETH02 | Ethiopia | *Mccp* | Positive for Mccp | *NVI, Ethiopia* | Microbial Culture |

Mccp- *M. capricolum* ssp*. capripneumoniae; * same strains from two different source*

*CIRAD-Centre International de Recherche en Agronomie pour le Développement; Vetmed- University of Veterinary Medicine; NVI-National Veterinary Laboratory;*
