# Supplementary material for: One-Step Multiplex RT-qPCR Assay for the Detection of Peste des petits ruminants virus, Capripoxvirus, Pasteurella multocida and Mycoplasma capricolum subspecies (ssp.) capripneumoniae
Source: PLoS One. 2016 Apr 28;11(4):e0153688. doi: 10.1371/journal.pone.0153688 (PMC4849753; doi:10.1371/journal.pone.0153688)
Supplement: S5 Table — (DOC) [file pone.0153688.s005.doc]

**Table S5:** Details of the non-specific pathogens tested using the one-step multiplex RT-qPCR

| **S No** | **Sample ID** | **Origin** | **Species/strain** | **Multiplex result & Detected pathogen(s)** | **Received from** | **Sample type** |
| --- | --- | --- | --- | --- | --- | --- |
|  | PG2\M agalactiae* | Spain | *M. agalactiae* PG2 (type strain) | Negative | CIRAD, France | Microbial Culture |
|  | p7-50\M leachii\PG50\Australia | Australia | *M. leachii* PG50 (type strain) | Negative | CIRAD, France | Microbial Culture |
|  | KS1\Mputrefaciens | USA | *M. putrefaciens* KS1 (type strain) | Negative | CIRAD, France | Microbial Culture |
|  | p7-3\Mmc"capri"\PG3\* | Turkey | *M. mycoides* ssp. *capri* PG3 (type strain) | Negative | CIRAD, France | Microbial Culture |
|  | n-c-l\Mmc"capri"\N108\NG | Nigeria | *M. mycoides* ssp. *capri* N108 (field strain) | Negative | CIRAD, France | Microbial Culture |
|  | Y6\Mmc"LC"\Y goat\Austr | Australia | *M. mycoides* ssp. *capri* Y-goat (reference strain) | Negative | CIRAD, France | Microbial Culture |
|  | 7302\Mmc"LC"\7302\FR | France | *M. mycoides* ssp*. capri* 7302 (field strain) | Negative | CIRAD, France | Microbial Culture |
|  | Apada\MmmSC\Afade\CM | Cameroon | *M. mycoides* ssp. *mycoides* Afade (field strain) | Negative | CIRAD, France | Microbial Culture |
|  | 94157\Mcc\94157\ET | Ethiopia | *M. capricolum* ssp. *capricolum* 94157 (field strain) | Negative | CIRAD, France | Microbial Culture |
|  | 7714\Mcc\7714\FR | France | *M. capricolum* ssp. *capricolum* 7714 (field strain) | Negative | CIRAD, France | Microbial Culture |
|  | CK\Mcc\C.kid\USA | USA | *M. capricolum* ssp. *capricolum* California kid (type strain) | Negative | CIRAD, France | Microbial Culture |
|  | *M. agalactiae PG2^T^** | Spain | *M. agalactiae* PG2 (type strain) | Negative | Vetmed, Vienna | Microbial Culture |
|  | *M. bovis PG45^T^* | USA | *M. bovis* PG45 (type strain) | Negative | Vetmed, Vienna | Microbial Culture |
|  | *M. capricolum ssp. capricolum California kid^T^* | USA | *M. capricolum* ssp*. capricolum* California kid (type strain) | Negative | Vetmed, Vienna | Microbial Culture |
|  | *M. conjunctivae HRC/581^T^* | USA | *M. conjunctivae* HRC/581 (type strain) | Negative | Vetmed, Vienna | Microbial Culture |
|  | *M. mycoides ssp. capri PG3^T^** | Turkey | *M. mycoides* ssp. *capri* PG3 (type strain) | Negative | Vetmed, Vienna | Microbial Culture |
|  | *M. ovipneumoniae Y98^T^* | Australia | *M. ovipneumoniae* Y98 (type strain) | Negative | Vetmed, Vienna | Microbial Culture |
|  | *ORF Virus D1701* | Germany | *NA* | Negative | HSL-AGES,Austria | Cell culture |
|  | *BRSV* | France | *NA* | Negative | TNVS, France | Cell culture |
|  | *Parainfluenzavirus 3* |  | *NA* | Negative | Vetmed, Vienna | Viral isolate |

** same strains from two different sources; CIRAD-Centre International de Rechercheen Agronomie pour le Développement; Vetmed- University of Veterinary Medicine; TNVS- Toulouse national veterinary school; HSL-AGES- High Security Laboratory, Austrian Agency for Health and Food Safety, Moedling.*
