# Supplementary material for: One-Step Multiplex RT-qPCR Assay for the Detection of Peste des petits ruminants virus, Capripoxvirus, Pasteurella multocida and Mycoplasma capricolum subspecies (ssp.) capripneumoniae
Source: PLoS One. 2016 Apr 28;11(4):e0153688. doi: 10.1371/journal.pone.0153688 (PMC4849753; doi:10.1371/journal.pone.0153688)
Supplement: S6 Table — (DOC) [file pone.0153688.s006.doc]

**Table S6: Details of the TNA samples extracted from different field samples collected from Goat and Sheep showing the respiratory infections (mainly suspected for PPRV) and results on testing by one-step multiplex RT-qPCR**

| **S No** | **Sample ID** | **Origin** | **Received from** | **Sample type** | **Multiplex result & Detected pathogen(s)** | **Result by confirmatory test** | **Lineage/Genotype for PPRV** |
| --- | --- | --- | --- | --- | --- | --- | --- |
|  | CIV\09_01P | Côte d’Ivoire(CIV) | LCPA, Côte d’Ivoire | Tissue | Positive for PPRV | Positive | II |
|  | CIV\09_02P | Côte d’Ivoire(CIV) | LCPA, Côte d’Ivoire | Tissue | Positive for PPRV | Positive | II |
|  | CIV\09_02P | Côte d’Ivoire(CIV) | LCPA, Côte d’Ivoire | Tissue | Positive for PPRV | Positive | II |
|  | CIV\09_OVP | Côte d’Ivoire(CIV) | LCPA, Côte d’Ivoire | Tissue | Positive for PPRV | Positive | II |
|  | CIV\12_01P | Côte d’Ivoire(CIV) | LCPA, Côte d’Ivoire | Tissue | Positive for PPRV | Positive | II |
|  | CIV\12_02P | Côte d’Ivoire(CIV) | LCPA, Côte d’Ivoire | Tissue | Positive for PPRV | Positive | II |
|  | CIV\CH1 | Côte d’Ivoire(CIV) | LCPA, Côte d’Ivoire | Tissue | Positive for PPRV | Positive | II |
|  | CIV\CH2 | Côte d’Ivoire(CIV) | LCPA, Côte d’Ivoire | Tissue | Positive for PPRV | Positive | II |
|  | GHA\10_AM | Ghana | CVL, Ghana | Tissue | Positive for PPRV | Positive | II |
|  | GHA\10_NK1 | Ghana | CVL, Ghana | Tissue | Positive for PPRV | Positive | II |
|  | GHA\10_TM1 | Ghana | CVL, Ghana | Tissue | Positive for PPRV | Positive | II |
|  | GHA\10_TM2 | Ghana | CVL, Ghana | Tissue | Positive for PPRV | Positive | II |
|  | KN01\2011 | Kenya | UN, Kenya | Tissue | Positive for PPRV | Positive | III |
|  | KN03\2011 | Kenya | UN, Kenya | MLN | Positive for PPRV | Positive | III |
|  | CAM\10_01 | Cameroon | LANAVET, Cameroon | Tissue | Positive for PPRV | Positive | IV |
|  | CAM\10_04 | Cameroon | LANAVET, Cameroon | Tissue | Positive for PPRV | Positive | IV |
|  | CAM\10_19 | Cameroon | LANAVET, Cameroon | Tissue | Positive for PPRV | Positive | IV |
|  | CAM\10_28 | Cameroon | LANAVET, Cameroon | Tissue | Positive for PPRV | Positive | IV |
|  | DRC22\2012 | DRC | LVK, DRC | Tissue | Positive for PPRV | Positive | IV |
|  | DRC23\2012 | DRC | LVK, DRC | Tissue | Positive for PPRV | Positive | IV |
|  | DRC24\2012 | DRC | LVK, DRC | Tissue | Positive for PPRV | Positive | IV |
|  | DRC25\2012 | DRC | LVK, DRC | Tissue | Positive for PPRV | Positive | IV |
|  | DRC26\2012 | DRC | LVK, DRC | Tissue | Positive for PPRV | Positive | IV |
|  | DRC27\2012 | DRC | LVK, DRC | Tissue | Positive for PPRV | Positive | IV |
|  | DRC28\2012 | DRC | LVK, DRC | Tissue | Positive for PPRV | Positive | IV |
|  | ETH\10_02 | Ethiopia | NVI, Ethiopia | Tissue | Positive for PPRV | Positive | IV |
|  | ETH\10_04 | Ethiopia | NVI, Ethiopia | Tissue | Positive for PPRV | Positive | IV |
|  | ETH\10_10 | Ethiopia | NVI, Ethiopia | Tissue | Positive for PPRV | Positive | IV |
|  | Panvac_Eth_13 | Ethiopia | PANVAC, Ethiopia | Tissue | Positive for PPRV | Positive | IV |
|  | Panvac_Eth_16 | Ethiopia | PANVAC, Ethiopia | Tissue | Positive for PPRV | Positive | IV |
|  | Panvac_Eth_21 | Ethiopia | PANVAC, Ethiopia | Tissue | Positive for PPRV | Positive | IV |
|  | Panvac_Eth_39 | Ethiopia | PANVAC, Ethiopia | Tissue | Positive for PPRV | Positive | IV |
|  | PPR\33203\ control RNA^γ^ | Ethiopia | NAHDIC, Ethiopia | Swab | Positive for PPRV | Positive | IV |
|  | PPR\33204\11102013\Demke^γ^ | Ethiopia | NAHDIC, Ethiopia | Swab | Positive for PPRV | Positive | IV |
|  | TUK\11-02 | Turkey | PVCRI, Turkey | Tissue | Positive for PPRV | Positive | IV |
|  | TUK\11-04 | Turkey | PVCRI, Turkey | Tissue | Positive for PPRV | Positive | IV |
|  | TUK\11-07 | Turkey | PVCRI, Turkey | Tissue | Positive for PPRV | Positive | IV |
|  | TUK\11-10 | Turkey | PVCRI, Turkey | Tissue | Positive for PPRV | Positive | IV |
|  | BKF03/201404 | Burkina Faso | LNE, Burkina Faso | Swab | Negative | Negative | NA |
|  | BKF12/201404 | Burkina Faso | LNE, Burkina Faso | Swab | Negative | Negative | NA |
|  | KN13\2011 | Kenya | UN, Kenya | Tissue | Negative | Negative | NA |
|  | KN15\2011 | Kenya | UN, Kenya | Tissue | Negative | Negative | NA |
|  | KN19\2011 | Kenya | UN, Kenya | Swab | Positive for PM | Positive | NA |
|  | TUK\11-08 | Turkey | PVCRI, Turkey | Tissue | Negative | Negative | NA |
|  | TUK\11-09 | Turkey | PVCRI, Turkey | Tissue | Negative | Negative | NA |
|  | BKF02/201404 | Burkina Faso | LNE, Burkina Faso | Swab | Positive for PPRV and PM | Positive | ND |
|  | BKF03/201404 | Burkina Faso | LNE, Burkina Faso | Swab | Negative | Negative | NA |
|  | BKF04/201404 | Burkina Faso | LNE, Burkina Faso | Swab | Positive for PM | Positive | NA |
|  | BKF05/201404 | Burkina Faso | LNE, Burkina Faso | Swab | Positive for PPRV and PM | Positive | ND |
|  | BKF06/201404 | Burkina Faso | LNE, Burkina Faso | Swab | Positive for PM | Positive | NA |
|  | BKF07/201404 | Burkina Faso | LNE, Burkina Faso | Swab | Positive for PPRV | Positive | ND |
|  | BKF08/201404 | Burkina Faso | LNE, Burkina Faso | Swab | Negative | Positive | NA |
|  | BKF09/201404 | Burkina Faso | LNE, Burkina Faso | Swab | Negative | Positive | NA |
|  | BKF10/201404 | Burkina Faso | LNE, Burkina Faso | Swab | Positive for PPRV and PM | Positive | ND |
|  | BKF11/201404 | Burkina Faso | LNE, Burkina Faso | Swab | Positive for PPRV | Positive | ND |
|  | BKF12/201404 | Burkina Faso | LNE, Burkina Faso | Swab | Negative | Negative | NA |
|  | BKF13/201404 | Burkina Faso | LNE, Burkina Faso | Swab | Positive for PPRV | Positive | ND |
|  | BKF14/201404 | Burkina Faso | LNE, Burkina Faso | Swab | Positive for PPRV | Positive | ND |

^γ-^ Received extracted RNA; *NA- Not applicable; ND- Not Done*

*LCPA- Laboratoire central de pathologie animale, LANADA; CVL- Central veterinary laboratorie; LANAVET - Laboratoire national vétérinaire;* LVK, DRC- *Laboratoire Vétérinaire de Kinshasa, Democratic republic of Congo; NVI- National Veterinary Institute; PANVAC- Pan-African Veterinary Vaccine Centre; NAHDIC- National Animal Health Diagnostic and Investigation Center; UN- University of Nairobi;* PVCRI- *Pendik Veterinary Control and Research Institute; LNE-Laboratorie National d’Elevage.*
