# Supplementary material for: One-Step Multiplex RT-qPCR Assay for the Detection of Peste des petits ruminants virus, Capripoxvirus, Pasteurella multocida and Mycoplasma capricolum subspecies (ssp.) capripneumoniae
Source: PLoS One. 2016 Apr 28;11(4):e0153688. doi: 10.1371/journal.pone.0153688 (PMC4849753; doi:10.1371/journal.pone.0153688)
Supplement: S7 Table — (DOC) [file pone.0153688.s007.doc]

**Table S7: Details of the DNA samples extracted from different pathological samples collected from Goat and Sheep showing the symptoms of respiratory infections and results on testing by one-step multiplex RT-qPCR**

| **S No** | **Sample ID** | **Origin** | **Received from** | **Sample type** | **Multiplex result & Detected pathogen(s)** | **Result by confirmatory test** | **Lineage/**  **genotype** |
| --- | --- | --- | --- | --- | --- | --- | --- |
|  | Metekei 7_15/11/2010_Sheep | Ethiopia | NVI, Ethiopia | Tissue | Positive for CaPV | Positive | GTPV |
|  | Wonji S_SS_09/11/2011_Sheep | Ethiopia | NVI, Ethiopia | Tissue | Positive for CaPV | Positive | GTPV |
|  | NVIPOX_Goat_01/10/2011 | Ethiopia | NVI, Ethiopia | Tissue | Positive for CaPV | Positive | GTPV |
|  | TFY_Oct_2014_Amhra13 | Ethiopia | NVI, Ethiopia | Tissue | Positive for CaPV | Positive | GTPV |
|  | TFY_Oct_2014_Oromo14 | Ethiopia | NVI, Ethiopia | Tissue | Positive for CaPV | Positive | GTPV |
|  | TFY_Oct_2014_Oromo15 | Ethiopia | NVI, Ethiopia | Tissue | Positive for CaPV | Positive | GTPV |
|  | TFY_Oct_2014_Afar17 | Ethiopia | NVI, Ethiopia | Tissue | Positive for CaPV | Positive | GTPV |
|  | TFY_Oct_2014_Afar18 | Ethiopia | NVI, Ethiopia | Tissue | Positive for CaPV | Positive | GTPV |
|  | Kitengala/O58/2011 | Kenya | CVL, Kenya | Tissue | Positive for CaPV | Positive | GTPV |
|  | Kitengala/O59/2011 | Kenya | CVL, Kenya | Tissue | Positive for CaPV | Positive | GTPV |
|  | Kiambu/G143/2009 | Kenya | CVL, Kenya | Tissue | Positive for CaPV | Positive | GTPV |
|  | MOG/SP/T1 | Mongolia | IVM, Mongolia | Tissue | Positive for CaPV | Positive | SPPV |
|  | MOG/SP/T2 | Mongolia | IVM, Mongolia | Tissue | Positive for CaPV | Positive | SPPV |
|  | MOG/SP/T3 | Mongolia | IVM, Mongolia | Tissue | Positive for CaPV | Positive | SPPV |
|  | MOG/GP/T4 | Mongolia | IVM, Mongolia | Tissue | Positive for CaPV | Positive | GTPV |
|  | MOG/GP/T5 | Mongolia | IVM, Mongolia | Tissue | Positive for CaPV | Positive | GTPV |
|  | MOG/GP/T6 | Mongolia | IVM, Mongolia | Tissue | Positive for CaPV | Positive | GTPV |
|  | LP Ebolowa 091/2014 | Cameroon | LANAVET, Cameroon | Tissue | Positive for PM | Positive | ND |
|  | LP Ebolowa 091/2014 | Cameroon | LANAVET, Cameroon | Tissue | Negative/None | ND | NA |
|  | PonctionVessie 091/2014 | Cameroon | LANAVET, Cameroon | Tissue | Negative/None | ND | NA |
|  | Sérum Ebolowa 091/2014 | Cameroon | LANAVET, Cameroon | Tissue | Negative/None | ND | NA |
|  | Poumon Ebolowa 091/2014 | Cameroon | LANAVET, Cameroon | Tissue | Negative/None | ND | NA |
|  | Sérum Mfou 088/2014 | Cameroon | LANAVET, Cameroon | Tissue | Negative/None | ND | NA |

*NVI- National Veterinary Institute, Ethiopia; CVL- Central Veterinary laboratories, Kenya; IVM- Institute of Veterinary Medicine, Ulan Bator, Mongolia; LANAVET - Laboratoire national vétérinaire, Cameroon*
