# Supplementary material for: One-Step Multiplex RT-qPCR Assay for the Detection of Peste des petits ruminants virus, Capripoxvirus, Pasteurella multocida and Mycoplasma capricolum subspecies (ssp.) capripneumoniae
Source: PLoS One. 2016 Apr 28;11(4):e0153688. doi: 10.1371/journal.pone.0153688 (PMC4849753; doi:10.1371/journal.pone.0153688)
Supplement: S8 Table — (DOC) [file pone.0153688.s008.doc]

**Table S8: Details of the DNA samples extracted from different pathological samples collected from cattle showing the symptoms of respiratory infections and results on testing by one-step multiplex RT-qPCR**

| **S No** | **Sample ID** | **Origin** | **Received from** | **Sample type** | **Multiplex result & Detected pathogen(s)** | **Result by confirmatory test** | **Lineage/**  **genotype** |
| --- | --- | --- | --- | --- | --- | --- | --- |
|  | Adama 1_SB_4/10/2011_Cattle | Ethiopia | NVI, Ethiopia | Tissue | Positive for CaPV | Positive | LSDV |
|  | Adama 2_MS_4/10/2011_Cattle | Ethiopia | NVI, Ethiopia | Tissue | Positive for CaPV | Positive | LSDV |
|  | Mojo  3_Bh_09/11/2011_Cattle | Ethiopia | NVI, Ethiopia | Tissue | Positive for CaPV | Positive | LSDV |
|  | Mojo 4_AD_09/11/2011_Cattle | Ethiopia | NVI, Ethiopia | Tissue | Positive for CaPV | Positive | LSDV |
|  | Wonji 6_AT_21/11/2011_cattle | Ethiopia | NVI, Ethiopia | Tissue | Positive for CaPV | Positive | LSDV |
|  | Wonji 6_AT_21/11/2011_cattle | Ethiopia | NVI, Ethiopia | Tissue | Positive for CaPV | Positive | LSDV |
|  | LSDV Massalamia P4 | Sudan | CVRL, Sudan | Tissue | Positive for CaPV | Positive | LSDV |
|  | LSDV Sinnar | Sudan | CVRL, Sudan | Tissue | Positive for CaPV | Positive | LSDV |
|  | LSDV Massalamia | Sudan | CVRL, Sudan | Tissue | Positive for CaPV | Positive | LSDV |
|  | LSDV Sudan | Sudan | CVRL, Sudan | Tissue | Positive for CaPV | Positive | LSDV |
|  | TFY_Oct_2014_01 | Ethiopia | NAHDIC, Ethiopia | Tissue | Positive for CaPV | Positive | LSDV |
|  | TFY_Oct_2014_02 | Ethiopia | NAHDIC, Ethiopia | Tissue | Positive for CaPV | Positive | LSDV |
|  | TFY_Oct_2014_03 | Ethiopia | NAHDIC, Ethiopia | Tissue | Positive for CaPV | Positive | LSDV |
|  | TFY_Oct_2014_04 | Ethiopia | NAHDIC, Ethiopia | Tissue | Positive for CaPV | Positive | LSDV |
|  | TFY_Oct_2014_05 | Ethiopia | NAHDIC, Ethiopia | Tissue | Positive for CaPV | Positive | LSDV |
|  | TFY_Oct_2014_06 | Ethiopia | NAHDIC, Ethiopia | Tissue | Positive for CaPV | Positive | LSDV |
|  | TFY_Oct_2014_07 | Ethiopia | NAHDIC, Ethiopia | Tissue | Positive for CaPV | Positive | LSDV |
|  | TFY_Oct_2014_08 | Ethiopia | NAHDIC, Ethiopia | Tissue | Positive for CaPV | Positive | LSDV |
|  | TFY_Oct_2014_09 | Ethiopia | NAHDIC, Ethiopia | Tissue | Positive for CaPV | Positive | LSDV |
|  | TFY_Oct_2014_10 | Ethiopia | NAHDIC, Ethiopia | Tissue | Positive for CaPV | Positive | LSDV |
|  | TFY_Oct_2014_11 | Ethiopia | NAHDIC, Ethiopia | Tissue | Positive for CaPV | Positive | LSDV |
|  | TFY_Oct_2014_12 | Ethiopia | NAHDIC, Ethiopia | Tissue | Positive for CaPV | Positive | LSDV |
|  | TFY_Oct_2014_16 | Ethiopia | NAHDIC, Ethiopia | Tissue | Positive for CaPV | Positive | LSDV |
|  | TFY_Oct_2014_19 | Ethiopia | NAHDIC, Ethiopia | Tissue | Positive for CaPV | Positive | LSDV |
|  | TFY_Oct_2014_20 | Ethiopia | NAHDIC, Ethiopia | Tissue | Positive for CaPV | Positive | LSDV |
|  | TFY_Oct_2014_21 | Ethiopia | NAHDIC, Ethiopia | Tissue | Positive for CaPV | Positive | LSDV |
|  | TFY_Oct_2014_22 | Ethiopia | NAHDIC, Ethiopia | Tissue | Positive for CaPV | Positive | LSDV |
|  | TFY_Oct_2014_23 | Ethiopia | NAHDIC, Ethiopia | Tissue | Positive for CaPV | Positive | LSDV |
|  | DRC01_FP_2014 | DRC | VLK, DRC | Tissue | Negative/None | ND | NA |
|  | DRC02_FP_2014 | DRC | VLK, DRC | Tissue | Negative/None | ND | NA |
|  | DRC03_FP_2014 | DRC | VLK, DRC | Tissue | Negative/None | ND | NA |
|  | DRC04_FP_2014 | DRC | VLK, DRC | Tissue | Negative/None | ND | NA |
|  | DRC05_FP_2014 | DRC | VLK, DRC | Tissue | Negative/None | ND | NA |
|  | DRC06_FP_2014 | DRC | VLK, DRC | Tissue | Positive for CaPV | Positive | LSDV |
|  | DRC07_FP_2014 | DRC | VLK, DRC | Tissue | Negative/None | ND | NA |
|  | DRC08_FP_2014 | DRC | VLK, DRC | Tissue | Negative/None | ND | NA |
|  | DRC09_FP_2014 | DRC | VLK, DRC | Tissue | Negative/None | ND | NA |
|  | DRC10_FP_2014 | DRC | VLK, DRC | Tissue | Negative/None | ND | NA |
|  | DRC11_FP_2014 | DRC | VLK, DRC | Tissue | Negative/None | ND | NA |
|  | DRC12_FP_2014 | DRC | VLK, DRC | Tissue | Negative/None | ND | NA |
|  | DRC13_FP_2014 | DRC | VLK, DRC | Tissue | Negative/None | ND | NA |
|  | DRC14_FP_2014 | DRC | VLK, DRC | Tissue | Negative/None | ND | NA |
|  | DRC15_FP_2014 | DRC | VLK, DRC | Tissue | Negative/None | ND | NA |
|  | DRC16_FP_2014 | DRC | VLK, DRC | Tissue | Negative/None | ND | NA |
|  | DRC17_FP_2014 | DRC | VLK, DRC | Tissue | Negative/None | ND | NA |
|  | DRC18_FP_2014 | DRC | VLK, DRC | Tissue | Negative/None | ND | NA |
|  | DRC19_FP_2014 | DRC | VLK, DRC | Tissue | Negative/None | ND | NA |
|  | DRC20_FP_2014 | DRC | VLK, DRC | Tissue | Negative/None | ND | NA |
|  | DRC21_FP_2014 | DRC | VLK, DRC | Tissue | Negative/None | ND | NA |
|  | DRC22_FP_2014 | DRC | VLK, DRC | Tissue | Negative/None | ND | NA |
|  | DRC23_FP_2014 | DRC | VLK, DRC | Tissue | Negative/None | ND | NA |
|  | DRC24_FP_2014 | DRC | VLK, DRC | Tissue | Positive for CaPV | Positive | LSDV |
|  | DRC25_FP_2014 | DRC | VLK, DRC | Tissue | Negative/None | ND | NA |
|  | DRC26_FP_2014 | DRC | VLK, DRC | Tissue | Negative/None | ND | NA |
|  | DRC27_FP_2014 | DRC | VLK, DRC | Tissue | Negative/None | ND | NA |
|  | DRC28_FP_2014 | DRC | VLK, DRC | Tissue | Negative/None | ND | NA |
|  | DRC29_FP_2014 | DRC | VLK, DRC | Tissue | Negative/None | ND | NA |
|  | DRC30_FP_2014 | DRC | VLK, DRC | Tissue | Negative/None | ND | NA |
|  | DRC31_FP_2014 | DRC | VLK, DRC | Tissue | Positive for CaPV | Positive | LSDV |
|  | DRC32_FP_2014 | DRC | VLK, DRC | Tissue | Negative/None | ND | NA |

*NVI- National Veterinary Institute, Ethiopia; CVRL-Central veterinary research Laboratories; NAHDIC- National Animal Health Diagnostic and Investigation Center; VLK***-** *Veterinary Laboratory of Kinshasa, Democratic Republic of Congo;*
