# Supplementary material for: One-Step Multiplex RT-qPCR Assay for the Detection of Peste des petits ruminants virus, Capripoxvirus, Pasteurella multocida and Mycoplasma capricolum subspecies (ssp.) capripneumoniae
Source: PLoS One. 2016 Apr 28;11(4):e0153688. doi: 10.1371/journal.pone.0153688 (PMC4849753; doi:10.1371/journal.pone.0153688)
Supplement: S9 Table — (DOC) [file pone.0153688.s009.doc]

**Table S9:** Primer sequences and reference used for the confirmation of four targeted pathogens-CaPV, PPRV, PM and Mccp

| ***Target Pathogen*** | ***Method*** | ***Forward primer*** | ***Reverse primer*** | ***Product Size*** | ***Reference*** |
| --- | --- | --- | --- | --- | --- |
| *CaPV* | Real time PCR | SBF-GGTGTAGTACGTATAAGATTATCGT  ATAGAAACAAGCCTTTA | Rev- AATTTCTTTCTCTGTTCCATTTG | 92 bp | [20] |
| *PPRV* | Classical PCR | NP3 TCTCGGAAATCGCCTCACAGACTG | NP4- CCTCCTCCTGGTCCTCCAGAATCT | 351 bp | [21] |
| *PM* | Classical PCR | KMT1_SP6 -GCTGTAAACGAACTCGCCC | KMT1_T7 ATCCGCTATTTACCCAGTGG | 460 bp | [22] |
| *Mccp* | Classical PCR | Mccp-spe-F-ATCATTTTTAATCCCTTCAAG | Mccp-spe-R-TACTATGAGTAATTATAATATATGCAA | 316 bp | [23] |
