# Supplementary material for: One-Step Multiplex RT-qPCR Assay for the Detection of Peste des petits ruminants virus, Capripoxvirus, Pasteurella multocida and Mycoplasma capricolum subspecies (ssp.) capripneumoniae
Source: PLoS One. 2016 Apr 28;11(4):e0153688. doi: 10.1371/journal.pone.0153688 (PMC4849753; doi:10.1371/journal.pone.0153688)
Supplement: S10 Table — (DOC) [file pone.0153688.s010.doc]

**Table S10:** Performance of the multiplex assay in detection of TNA of different targets (represented by Cq values) extracted using two different extraction kits (Roche and Qiagen).

Pathological samples with confirmed positivity of the targeted pathogens individually or in form of mixed infection were selected. Total nucleic acids were extracted using two different extraction kits and used for further amplification and detection using the multiplex one step RT-qPCR. The amplification pattern indicated the better performance of the RNeasy extraction kit (Qiagen).

| **Sample ID** | **Detected with pathogen(s)** | **CaPV-Cy5** | | **PPRV-HEX** | | **PM-FAM** | | **Mccp-TxR** | | **Sample type** |
| --- | --- | --- | --- | --- | --- | --- | --- | --- | --- | --- |
|  |  | **Roche** | **Qiagen** | **Roche** | **Qiagen** | **Roche** | **Qiagen** | **Roche** | **Qiagen** |  |
| C2_B291/2007 | Positive for CaPV | NA^$^ | 22.96^$^ | NA | NA | NA | NA | NA | NA | Tissue |
| Kiambu/G143/2009 | Positive for CaPV | NA^$^ | 17.27^$^ | NA | NA | NA | NA | NA | NA | Tissue |
| BKF13/201404 | Positive for PPRV | NA | NA | 27.00^¥^ | 26.16^¥^ | NA | NA | NA | NA | Swab |
| BKF14/201404 | Positive for PPRV | NA | NA | 27.00^$^ | 21.58^$^ | NA | NA | NA | NA | Swab |
| BKF04/201404 | Positive for PM | NA | NA | NA | NA | 25.10^$^ | 22.42^$^ | NA | NA | Swab |
| BKF06/201404 | Positive for PM | NA | NA | NA | NA | 29.82^$^ | 27.19^$^ | NA | NA | Swab |
| Unknown | Positive for CaPV and PPRV | 23.43^¥^ | 23.53^¥^ | NA^$^ | 27.50^$^ | NA | NA | NA | NA | Tissue |
| Unknown | Positive for CaPV and PPRV | 21.75^¥^ | 22.51^¥^ | NA^$^ | 26.96^$^ | NA | NA | NA | NA | Tissue |
| BKF02/201404 | Positive for PPRV and PM | NA | NA | 22.86^¥^ | 23.03^¥^ | 23.05^¥^ | 21.91^¥^ | NA | NA | Swab |
| Unknown | Positive for PPRV and PM | NA | NA | 25.58^$^ | 19.49^$^ | 25.77^*^ | 28.01^*^ | NA | NA | Tissue |

$- Better performance of Qiagen extraction kit indicated by no amplification (NA) or higher Cq observed in samples extracted using Roche kit; ¥-indicates equal performance by both the extraction kits with similar Cq values;

*- better performance of Roche extraction kit indicated by higher Cq value in sample extracted using Qiage kit (observed only in one sample)
