# Supplementary material for: One-Step Multiplex RT-qPCR Assay for the Detection of Peste des petits ruminants virus, Capripoxvirus, Pasteurella multocida and Mycoplasma capricolum subspecies (ssp.) capripneumoniae
Source: PLoS One. 2016 Apr 28;11(4):e0153688. doi: 10.1371/journal.pone.0153688 (PMC4849753; doi:10.1371/journal.pone.0153688)
Supplement: S11 Table — (DOC) [file pone.0153688.s011.doc]

**Table S11:** Comparison of performance of One-step multiplex RT-PCR assay for detection of PPRV with two previously described methods

Dilutions of PPRV RNA transcripts (control) and two different TNA extracts (TNA1 and TNA2) were amplified and detected using three real time PCR methods. The amplification pattern (represented by Cq values) overall indicated the similar performance of one-step RT-PCR multiplex along with the other two methods. However, method by Bao *et al.,* comparatively showed lower Cq values but detection efficiency at higher dilutions of transcripts and TNA was similar to that of one-step multiplex RT-PCR, whereas the method by Kwiatek *et al.,* was showing higher Cq values or no amplification.

|  | **One-step Multiplex RT-PCR** | **Bao *et al*., 2008** [26] | **Kwiatek *et al*., 2010** [27] |
| --- | --- | --- | --- |
| **Copy number of RNA Transcripts** |  |  |  |
| **50** | N/A | N/A | N/A |
| **100** | N/A | N/A | N/A |
| **200** | 32.51 | 32.53 | N/A |
| **300** | 31.10 | 31.33 | 35.11 |
| **400** | 31.03 | 31.56 | 29.07 |
| **500** | 30.51 | 30.36 | 28.94 |
| **5000** | 27.70 | 27.67 | 25.25 |
| **50000** | 24.37 | 24.65 | 21.97 |
| **Dilutions of TNA1** |  |  |  |
| **10^-4^** | 34.93 | 34.97 | N/A |
| **10^-3^** | 32.57 | 29.35 | 33.30 |
| **10^-2^** | 28.67 | 27.23 | 27.49 |
| **10^-1^** | 25.38 | 24.01 | 24.40 |
| **TNA1** | 21.60 | 20.78 | 21.06 |
| **Dilutions of TNA2** |  |  |  |
| **10^-3^** | 31.35 | 29.11 | 36.16 |
| **10^-2^** | 28.25 | 26.53 | 28.07 |
| **10^-1^** | 24.95 | 23.53 | 24.71 |
| **TNA2** | 21.16 | 20.26 | 21.23 |
